# Novel Imidazopyridine Derivatives Possess Anti-Tumor Effect on Human Castration-Resistant Prostate Cancer Cells

**DOI:** 10.1371/journal.pone.0131811

**Published:** 2015-06-29

**Authors:** Matthew A. Ingersoll, Anastesia S. Lyons, Sakthivel Muniyan, Napoleon D’Cunha, Tashika Robinson, Kyle Hoelting, Jennifer G. Dwyer, Xiu R. Bu, Surinder K. Batra, Ming-Fong Lin

**Affiliations:** 1 Department of Biochemistry and Molecular Biology, University of Nebraska Medical Center, Omaha, Nebraska, United States of America; 2 Department of Chemistry, Clark Atlanta University, Atlanta, Georgia, United States of America; 3 Department of Biological Sciences, Clark Atlanta University, Atlanta, Georgia, United States of America; 4 College of Pharmacy, University of Nebraska Medical Center, Omaha, Nebraska, United States of America; 5 Section of Urology, Department of Surgery, University of Nebraska Medical Center, Omaha, Nebraska, United States of America; 6 Laboratory for Electro-Optical Materials & NASA Center for High Performance Polymers and Composites, Clark Atlanta University, Atlanta, Georgia, United States of America; 7 Eppley Institute for Research in Cancer and Allied Diseases, University of Nebraska Medical Center, Omaha, Nebraska, United States of America; 8 College of Pharmacy, Kaohsiung Medical University, Kaohsiung, Taiwan, 807, ROC; Innsbruck Medical University, AUSTRIA

## Abstract

Prostate cancer (PCa) is the second leading cause of cancer-related death afflicting United States males. Most treatments to-date for metastatic PCa include androgen-deprivation therapy and second-generation anti-androgens such as abiraterone acetate and enzalutamide. However, a majority of patients eventually develop resistance to these therapies and relapse into the lethal, castration-resistant form of PCa to which no adequate treatment option remains. Hence, there is an immediate need to develop effective therapeutic agents toward this patient population. Imidazopyridines have recently been shown to possess Akt kinase inhibitory activity; thus in this study, we investigated the inhibitory effect of novel imidazopyridine derivatives HIMP, M-MeI, OMP, and EtOP on different human castration-resistant PCa cells. Among these compounds, HIMP and M-MeI were found to possess selective dose- and time-dependent growth inhibition: they reduced castration-resistant PCa cell proliferation and spared benign prostate epithelial cells. Using LNCaP C-81 cells as the model system, these compounds also reduced colony formation as well as cell adhesion and migration, and M-MeI was the most potent in all studies. Further investigation revealed that while HIMP primarily inhibits PCa cell growth via suppression of PI3K/Akt signaling pathway, M-MeI can inhibit both PI3K/Akt and androgen receptor pathways and arrest cell growth in the G2 phase. Thus, our results indicate the novel compound M-MeI to be a promising candidate for castration-resistant PCa therapy, and future studies investigating the mechanism of imidazopyridine inhibition may aid to the development of effective anti-PCa agents.

## Introduction

Prostate cancer (PCa) remains the most commonly diagnosed solid tumor and the second leading cause of cancer-related death in United States men, maintaining a need for new effective treatment options [1]. Currently, androgen-deprivation therapy (ADT) is the standard course of treatment for metastatic PCa, however, most PCa patients relapse within 1–3 years and develop castration-resistant (CR) PCa which is unresponsive to ADT [2,3,4]. In 2004, a combination of docetaxel and prednisone was shown to increase patient median survival by 2–3 months, making it the standard-of-care treatment for CR PCa [5]. Recently, the FDA has approved additional compounds such as novel taxane chemotherapeutic cabazitaxel [6], androgen synthesis inhibitor abiraterone acetate [7], AR signaling inhibitor enzalutamide [8], immunotherapeutic sipuleucel-T [9], and bone micro-environment-targeted radiopharmaceutical alpharadin (Radium-223) for treating CR PCa [10]. However, these treatment options are only able to prolong survival by a few months and the average period of CR PCa patient survival remains less than two years [11]. Despite advancements in post-ADT treatment strategies, CR PCa remains an incurable disease; thus there is a great need for alternative therapeutic options.

While androgen insensitivity can be manifested in multiple ways; one proposed alternative mechanism is the increased activation of Akt signaling under androgen deprived conditions. Akt is known to regulate cell cycle, metabolism, angiogenesis, and cell survival in PCa and its activation may contribute to tumor resistance to ADT and anti-androgens [12,13]. One mechanism through which Akt may contribute to PCa survivability is via modulation of androgen receptor (AR) signaling. In addition to inducing cell growth, AR also has a role in regulating apoptosis. Upon phosphorylation of AR at Ser-210 and Ser-790 by Akt, AR-mediated apoptosis is suppressed. Through this mechanism, enhanced Akt activity in PCa may contribute to PCa survivability upon ADT [13]. Indeed, genetic loss and/or mutations in the phosphatidylinositol-3 kinase (PI3K)/Akt pathway that lead to signal deregulation may present in up-to 42% of primary prostate tumors and over 90% of metastatic tumors, making it a priority next-in-line therapeutic target [14]. Recently, investigations into imidazopyridines, a novel class of compounds containing aromatic aldehydes and a pyridine group, have demonstrated these compounds possess potent Akt kinase inhibitory activity [15–17]. Data shows these compounds have an anti-proliferative effect against CR PCa cells with the ability to simultaneously inhibit AR and PI3K/Akt/mTOR signaling pathways, making them promising therapeutic agents [18].

To investigate imidazopyridines’ efficacy for PCa therapy, the LNCaP progressive cell model, originally characterized in Lin et. al. *JBC* 1998, was used as the primary cell model in this study. LNCaP C-81 cells are androgen-independent (AI), express prostate-specific antigen (PSA) in the absence of androgens, and gain the ability to synthesize testosterone from cholesterol under steroid-reduced (SR) conditions [19–22]. C-81 cells also possess enhanced proliferation, ability to form colonies, and migratory potential [21,23]. Most Importantly, LNCaP C-81 cells retain AR expression and correspond to the expression of AR in the majority of PCa as well as advanced CR PCa [19]. This makes them a superior cell model for therapeutic studies when compared to many other PCa cell lines. Other cell lines selected for this study include MDA PCa2b-AI, PC-3, and RWPE1. Upon passage, MDA PCa2b cells behave similarly to LNCaP cells and shift from androgen-sensitive (AS) at low passage to AI at high passage. MDA PCa2b-AI (MDA-AI) cells also retain AR expression and possess enhanced tumorgenicity; this makes MDA-AI and LNCaP C-81 preferable cell models for studying prostate adenocarcinoma. Further, due to the ability of imidazopyridine derivatives to target both Akt and AR pathways, it is prudent to investigate the compounds’ effects on AR-negative PC-3 cells to determine their efficacy in cells which lack classic androgen signaling mechanisms. In addition, PC-3 cell lines are more representative of small-cell neuroendocrine carcinoma than more clinically predominant adenocarcinoma [24]; therefore this cell line should be used in conjunction with models such as LNCaP and MDA PCa2b cell lines to expand clinical utility. Finally, immortalized benign prostate epithelium RWPE1 cells act as a control to gauge the selectivity of the imidazopyridine derivative compounds. Thus our cell models clearly represent the majority of molecular events observed in clinical implementations of modern PCa therapies.

Our results demonstrate these imidazopyridine derivatives are able to suppress human PCa cell proliferation in a dose- and time-dependent manner. Importantly, compound M-MeI exhibited selective potency against CR PCa cell proliferation in comparison to benign prostate epithelial cells. Furthermore, this compound was also found to inhibit cell migration, adhesion, and *in vitro* tumorigenicity. Our data is the first to demonstrate the anti-tumor effect of novel imidazopyridine derivatives HIMP, M-MeI, OMP, and EtOP on CR PCa cells and indicates M-MeI to be a promising lead therapeutic agent for future studies.

## Materials and Methods

### Materials

**Fig 1:**
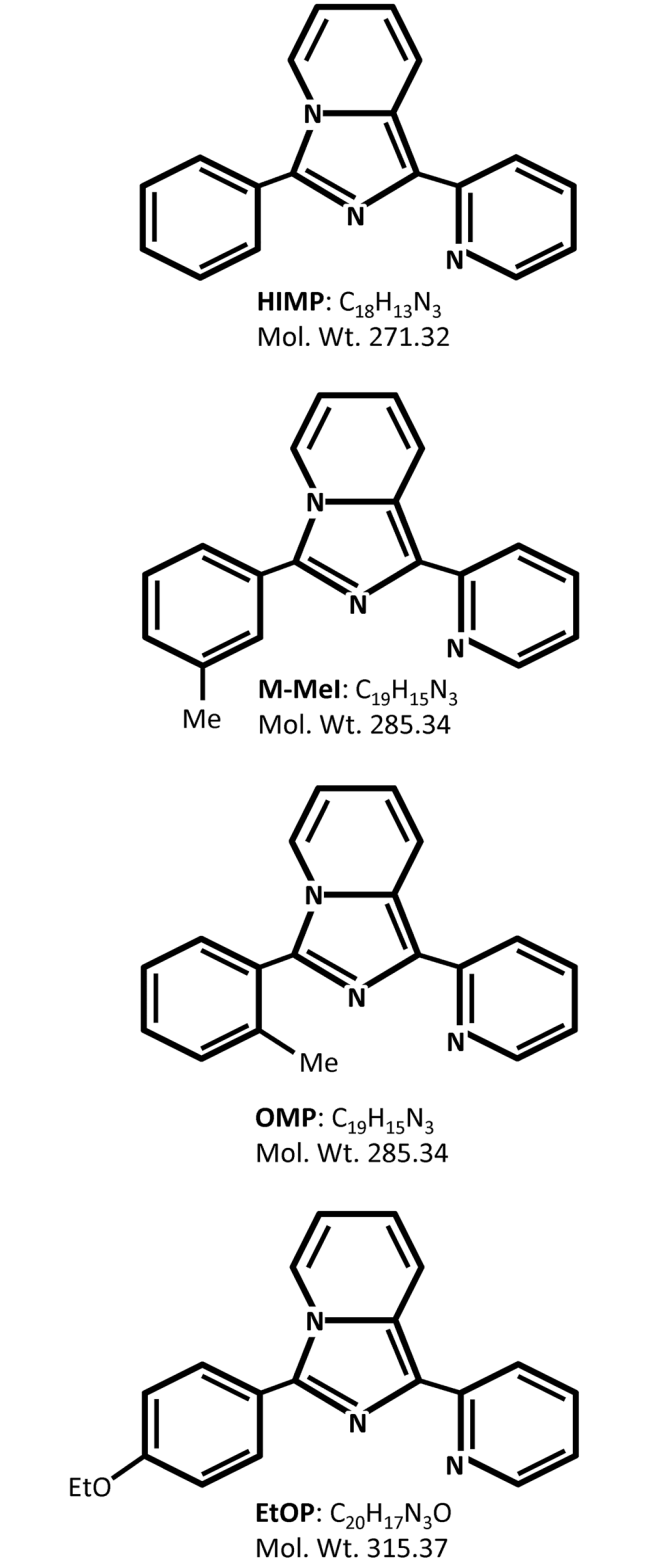
Structures of imidazopyridine derivatives. HIMP, 3-phenyl-1-(pyridine-2-yl)imidazo[1,5-*a*]pyridine; M-MeI, 1-(pyridine-2-yl)-3-(*m*-tolyl)imidazo[1,5-*a*]pyridine; OMP,1-(pyridine-2-yl)-3-(*o*-tolyl)imidazo[1,5-*a*]pyridine; EtOP, 3-(4-ethoxyphenyl)-1-(pyridine-2-yl)imidazo[1,5-*a*]pyridine.

RPMI 1640 medium, Keratinocyte SFM medium, gentamicin, and L-glutamine were purchased from Invitrogen (Carlsbad, CA). Fetal bovine serum (FBS) and charcoal-treated FBS were obtained from Atlanta Biologicals (Lawrenceville, GA). Molecular biology-grade agarose was procured from Fisher Biotech (Fair Lawn, NJ). Protein molecular weight standard markers, acrylamide, and Bradford protein assay kit were purchased from Bio-Rad (Hercules, CA). Polyclonal antibodies (Abs) recognizing all three isoforms of Shc protein (#29807, 1:4000) were purchased from Upstate (Lake Placid, NY). Anti-AR (#C1411, 1:400), anti-cyclin B_1_ (#K1907, 1:1000), anti-cyclin D_1_ (#A2712, 1:1000), anti-Bcl_XL_ (#F111, 1:1000), anti-Bax (#G241, 1:1000), anti-PCNA (#G261, 1:3000), anti-p53 (#K2607, 1:1000), anti-PSA (#E1812, 1:2000), anti-Survivin (#C271, 1:2000), and horseradish peroxidase-conjugated anti-mouse (#C2011, 1:5000), anti-rabbit (#D2910, 1:5000), anti-goat (#J0608, 1:5000) IgG Abs were all obtained from Santa Cruz Biotechnology (Santa Cruz, CA). Anti-phospho-Akt (Ser473) (#GA160, 1:1000), anti-Akt (#C1411, 1:2000), Anti-phospho-Stat5 (Y694) (#9351S, 1:4000), and anti-Stat5 (#9363, 1:2000) Abs were from Cell Signaling Technology (Beverly, MA). Anti-β-actin (#99H4842, 1:10000) Abs and 5α-dihydrotestosterone (DHT) were procured from Sigma (St. Louis, MO). Imidazopyridine derivatives HIMP (3-phenyl-1-(pyridine-2-yl)imidazo[1,5-*a*]pyridine), M-MeI (1-(pyridine-2-yl)-3-(*m*-tolyl)imidazo[1,5-*a*]pyridine), OMP (1-(pyridine-2-yl)-3-(*o*-tolyl)imidazo[1,5-*a*]pyridine), and EtOP (3-(4-ethoxyphenyl)-1-(pyridine-2-yl)imidazo[1,5-*a*]pyridine) were synthesized and provided by Dr. Xiu Bu as previously described [18,25] and their structures are shown in Fig 1. For ease of reading, chemical abbreviations are used throughout the text.

### Cell Culture

Human prostate carcinoma cell lines LNCaP, MDA PCa2b, PC-3, and immortalized benign prostate epithelial RWPE1 cells were originally obtained from the American Type Culture Collection (Rockville, MD, USA). LNCaP and PC-3 cells were routinely maintained in RPMI 1640 medium containing 5% FBS, 2 mM glutamine, and 50 μg/ml gentamicin [19,21]. MDA PCa2b cells were maintained in BRFF-HPC1 medium containing 20% FBS, 2 mM glutamine and 50 μg/ml gentamicin [26,27]. As reported previously, the LNCaP progressive cell model was established in which LNCaP cells at or below passage 33 are designated as C-33 and those at or greater than passage 81 as C-81. While C-33 cells are sensitive to androgen-induced growth, C-81 cells are AI, express higher basal levels of PSA, and gain the ability to synthesize testosterone from cholesterol [19–22]. Similar to the LNCaP model, upon passage, MDAPCa2b cells become AI and possess many biochemical properties of clinical CR PCa, including the expression of functional AR, PSA secretion, and rapid cell proliferation in androgen-deprived conditions [19,21,22,27,28]. RWPE1 cells were cultured in Keratinocyte-SFM supplemented with bovine pituitary extract (25 μg/ml) and recombinant epidermal growth factor (0.15 ng/ml) along with 50 μg/ml gentamicin.

To mimic conditions of clinical ADT, cells were maintained in SR conditions, i.e., phenol red-free RPMI 1640 medium containing 5% charcoal/dextran-treated FBS, 2 mM glutamine, and 50 μg/ml gentamicin plus 1 nM DHT. Imidazopyridine derivatives HIMP, M-MeI, OMP, and EtOP were dissolved in dimethyl sulfoxide (DMSO) at 20 mM stock concentrations, stored at -20°C and diluted as needed for experimental conditions in the respective medium.

### Cell Proliferation Assays

For cell proliferation experiments under regular conditions, LNCaP C-81 and MDA PCa2b-AI cells were seeded in regular culture medium and allowed to grow for 3 days, then changed to medium containing the respective compound and cultured for an additional 3 days. To determine cell proliferation under SR conditions, LNCaP C-81, MDA PCa2b-AI, PC-3, and RWPE1 cells were seeded in regular conditions and allowed to grow for 3 days. Cells were then steroid starved for 48 hours in SR medium and changed to fresh SR medium containing the respective compound, then cultured for an additional 3 days. Control groups received solvent DMSO alone. At the specified time point, cells were trypsinized and live cell numbers were counted via Trypan Blue Exclusion assay using a Cellometer Auto T4 Image-based cell counter (Nexcelom, MA, USA).

### Cell Growth Kinetic and Dosage Determinations

Dose-dependent assays were conducted on LNCaP C-81 cells in the same manner as cell proliferation assays and used medium containing 0, 1, 5, or 10 μM of specified compound. To determine the kinetic effect of HIMP and M-MeI on the growth of LNCaP C-81 cells, cells were seeded into six-well culture plates at a density of 2 x 10^3^ cells/cm^2^ and maintained in regular culture conditions for 3 days. Cells were then changed to SR medium and maintained for 2 days. One plate of attached cells was harvested and counted as day 0, and the remaining cells were changed to their respective treatment medium: Control (DMSO), HIMP, and M-MeI (10 μM). At each time point, cells were harvested for cell number counting and the remaining cells were fed with fresh media containing respective treatment compound. After cell number counting, cell lysates were prepared for Western blot analysis.

### Flow Cytometry Analysis

To determine the compounds’ effect on cell cycle, LNCaP C-81 cells were seeded in T25 flasks at a density of 2 x 10^3^ cells/cm^2^ in regular medium for 3 days, changed to SR medium for 48 hours, and then fed with fresh SR medium containing 10 μM of specified compound. One set of cells was harvested after 3, 5, and 7 days of treatment, respectively, by trypsinization. After cell number counting, an aliquot of cells was pelleted by centrifugation, resuspended in 70% ethanol, and incubated at 4°C for 30 minutes, then washed with PBS and spun down again by centrifugation. The DNA of ethanol-fixed cells was stained using Telford Reagent (PBS, pH 7.4, containing 0.1% Triton X-100, 0.1mM EDTA disodium salt, 0.05mg/ml RNase A (50 U/mg), and 50 mg/ml propidium iodide) at 4°C for 4 hours [29]. Determination of cell cycle distribution was carried out using a Becton-Dickinson fluorescence-activated cell sorter (FACSCalibur, Becton Dickinson, San Jose, CA, USA) at the UNMC Flow Cytometry Core Facility.

### Cell Adhesion Assay

To determine the effect of imidazopyridine derivatives on PCa cell adhesion to plastic-ware surfaces, LNCaP C-81 cells were suspended in 5% FBS 1640 RPMI medium containing 10 μM of respective compounds and incubated for 30 minutes. Cells were then plated in 6-well plates in triplicates at a density of 3x10^3^ cells/cm^2^ in respective treatment medium and incubated for an additional hour. Non-attached cells were carefully washed away and the remaining attached cells were stained with a 0.2% crystal violet solution containing 50% methanol. The total number of cells in five fields at 40x magnification per well were counted.

### Clonogenic and Soft Agar Colony Formation Assays

The clonogenic cell growth assay on the surface of plastic-wares was conducted as described previously [23,26]. Briefly, LNCaP C-81 cells were plated into 6-well plates under regular culture conditions at three densities: 20, 200, and 2,000 cells per well. Cells were incubated overnight, after which unattached cells were removed and those attached cells were fed with fresh regular medium containing 10 μM of treatment compound. Cells were grown for 9 days with a change of fresh medium every three days. On the 10^th^ day, the medium was removed and cells were washed with ice-cold HEPES-buffered saline, then attached cells were stained with a 0.2% crystal violet solution containing 50% methanol. The experiment was carried out in duplicate.

The effect of imidazopyridine derivatives on anchorage-independent growth of LNCaP C-81 cells was assessed by soft agar assay. Briefly, 5x10^4^ cells were seeded into a 0.25% agarose top layer with a base layer containing 0.3% agarose in 6-well plates. The day after seeding, cell clusters containing more than one cell were excluded from the study. Cells were then fed with 0.5 mL of fresh regular medium containing the respective compound every 3 days for 4 weeks. After the experimental period, colonies were stained with a 0.2% crystal violet solution containing 50% methanol and counted.

### Cell Migration Assay

To determine the effect of imidazopyridine derivatives on PCa cell mobility, LNCaP C-81 cell migration was assessed via Boyden chamber assay. Cells were plated at a density of 5 x 10^4^ cells into the upper chamber of 24-well plate transwell inserts. Medium containing 10 μM of treatment compound (solvent alone for control) was placed in both upper and lower chambers of the transwells. Cells were then incubated for 24 hours, after which they were stained with 0.2% crystal violet solution in 50% methanol, and cells remaining in the upper chamber were removed via cotton swab. Cells which had migrated through to the lower chamber were counted at 40x magnification under a microscope.

### Immunoblot Analysis

All cells were rinsed with ice-cold HEPES-buffered saline, pH 7.0, harvested via scraping, and lysed in ice-cold lysis buffer containing protease and phosphatase inhibitors. Total cellular lysates were prepared as previously described [19,30]. The protein concentration of the supernatant was determined using a Bio-Rad Bradford protein-assay. For immunoblotting, an aliquot of total cell lysate was electrophoresed on SDS-polyacrylamide gels (7.5%-12%). After being transferred to nitrocellulose membrane, membranes were blocked with 5% non-fat milk in Tris-buffered saline (TBS) containing 0.1% Tween-20 for 30 minutes at room temperature. Membranes were incubated with the corresponding primary Ab overnight at 4°C. Membranes were then rinsed and incubated with the appropriate secondary Ab for 60 minutes at room temperature. Proteins of interest were detected by an enhanced chemiluminescence (ECL) reagent kit and β-actin was used as a loading control.

### Statistical Analysis

Each set of experiments was conducted in duplicate or triplicate as specified in the figure legends, and experiments were repeated independently at least two or three times. The mean and standard error values of all results were calculated and two-tailed student-t test was used to determine significance of results. *p*<0.05 was considered statistically significant.

## Results

### Dose-Dependent Effect of Imidazopyridine Derivatives on CR PCa cell Proliferation

**Fig 2:**
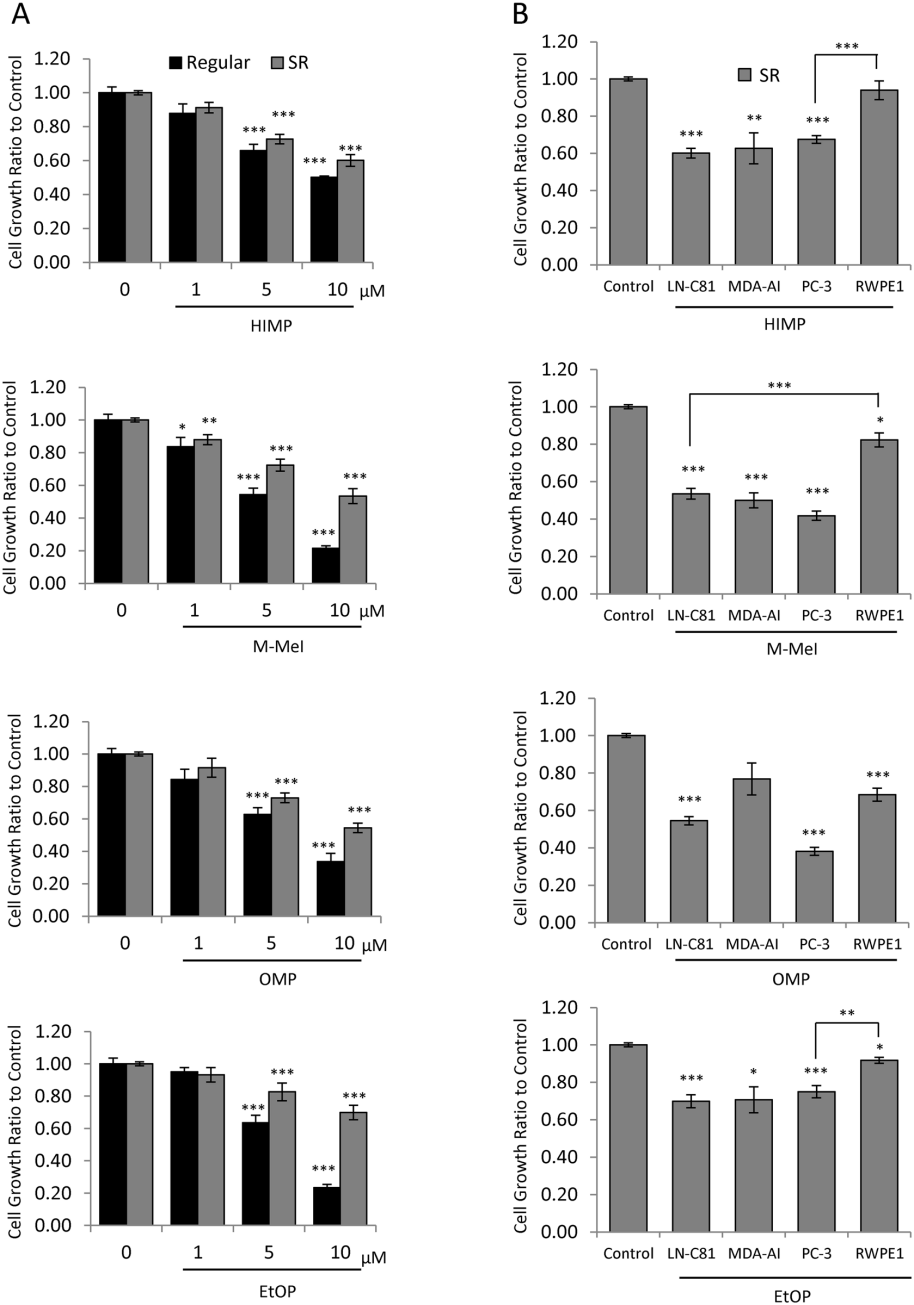
Effects of imidazopyridine derivatives on the proliferation of prostate epithelial cells. **(A)** Dosage effect of Imidazopyridine derivatives on LNCaP C-81 cells. Cells were plated in six-well plates at 2 x 10^3^ cells/cm^2^ in regular medium and grown for 72 hours. One set of cells was then fed with fresh regular medium containing 0,1,5, or 10 μM imidazopyridine derivatives with solvent alone for control and grown for an additional 72 hours. Another set of cells was first steroid starved in SR medium for 48 hours then treated with respective compounds in fresh SR media containing 1 nM DHT for 72 hours. All cells were trypsinized and live cell numbers were counted. The experiment was conducted in duplicate wells with 3 sets of independent experiments. The results presented are mean ± SE; n = 2x3. *p<0.05 **p<0.005 ***p<0.0005. **(B)** Effects of imidazopyridine derivatives on the growth of various PCa cells and immortalized prostate epithelial cells. All cells were plated in six-well plates at the noted density in their respective medium for three days, steroid-starved for two days, then fed with fresh SR medium with 1 nM DHT containing 10 μM imidazopyridine derivatives and grown for three additional days. Cells were trypsinized and live cell number was counted. LNCaP C-81–2 x 10^3^ cells/cm^2^, MDA PCab2b AI—3 x 10^3^ cells/cm^2^, PC-3–2 x 10^3^ cells/cm^2^, RWPEI– 7.5 x10^3^ cells/cm^2^. All experiments were performed in triplicate wells with 3 sets of independent experiments. Results presented are mean ± SE; n = 3x3. *p<0.05; **p<0.001; ***p<0.0001.

LNCaP C-81 cells exhibit many biochemical properties as seen in clinical CR PCa, including functional AR expression, AI PSA secretion, and proliferation with intracrine growth regulation [19,21–23] and thus were used as the primary cell model system for testing imidazopyridine compounds. Initially, the dose-dependent effects of HIMP, M-MeI, OMP, and EtOP on LNCaP C-81 cells were tested under regular culture conditions. Cells were treated with 0–10 μM of each compound for 72 hours and cell growth was analyzed via Trypan Blue exclusion assay. Under regular culture conditions, dose-dependent inhibition of cell proliferation was observed for all compounds with estimated IC_50_ values of 6.1 μM (M-MeI), 6.6 μM (EtOP), 7.3 μM (OMP), and 9.3 μM (HIMP) (Fig 2A).

We then examined the effect of the compounds in SR conditions, mimicking ADT conditions. The compounds inhibited cell growth following the dosage response with estimated IC_50_ values of 10.2 μM (M-MeI), 10.5 μM (OMP), 11.6 μM (HIMP), and 16.0 μM (EtOP) (Fig 2A). Interestingly, M-MeI had the greatest inhibitory activity under both growth conditions. Though EtOP had comparable inhibition to M-MeI in regular conditions, it had the least effect under SR conditions. HIMP and OMP were also shown to be less effective than M-MeI under both treatment conditions.

### Selective Anti-Proliferative Effect of Compounds on PCa vs. Immortalized Normal Prostate Epithelial Cells

The suppressive effect of each inhibitor on proliferation was investigated using a panel of cancerous and benign prostate epithelial cell lines. AI PCa cells including AR-positive LNCaP C-81 and MDA PCa2b-AI as well as AR-negative PC-3 cells were chosen as representatives of advanced CR PCa. RWPE1 cells, an immortalized benign prostate epithelial cell line, were used to determine the compounds’ selectivity. After three days of 10 μM treatment under SR conditions, HIMP, M-MeI, and EtOP all displayed selective inhibition of proliferation of cancerous cells with significantly less effect on non-cancerous RWPE1 cells (Fig 2B). Though OMP was effective against C-81 and PC-3 cells, it was comparatively potent against RWPE1 cells. Overall, the results show HIMP and M-MeI were the most selective, inhibiting PCa cell growth significantly more than RWPE1 cells with M-MeI displaying greater inhibition in all cell lines analyzed.

### Suppression of PCa Tumorigenicity by Imidazopyridine Derivatives

**Fig 3:**
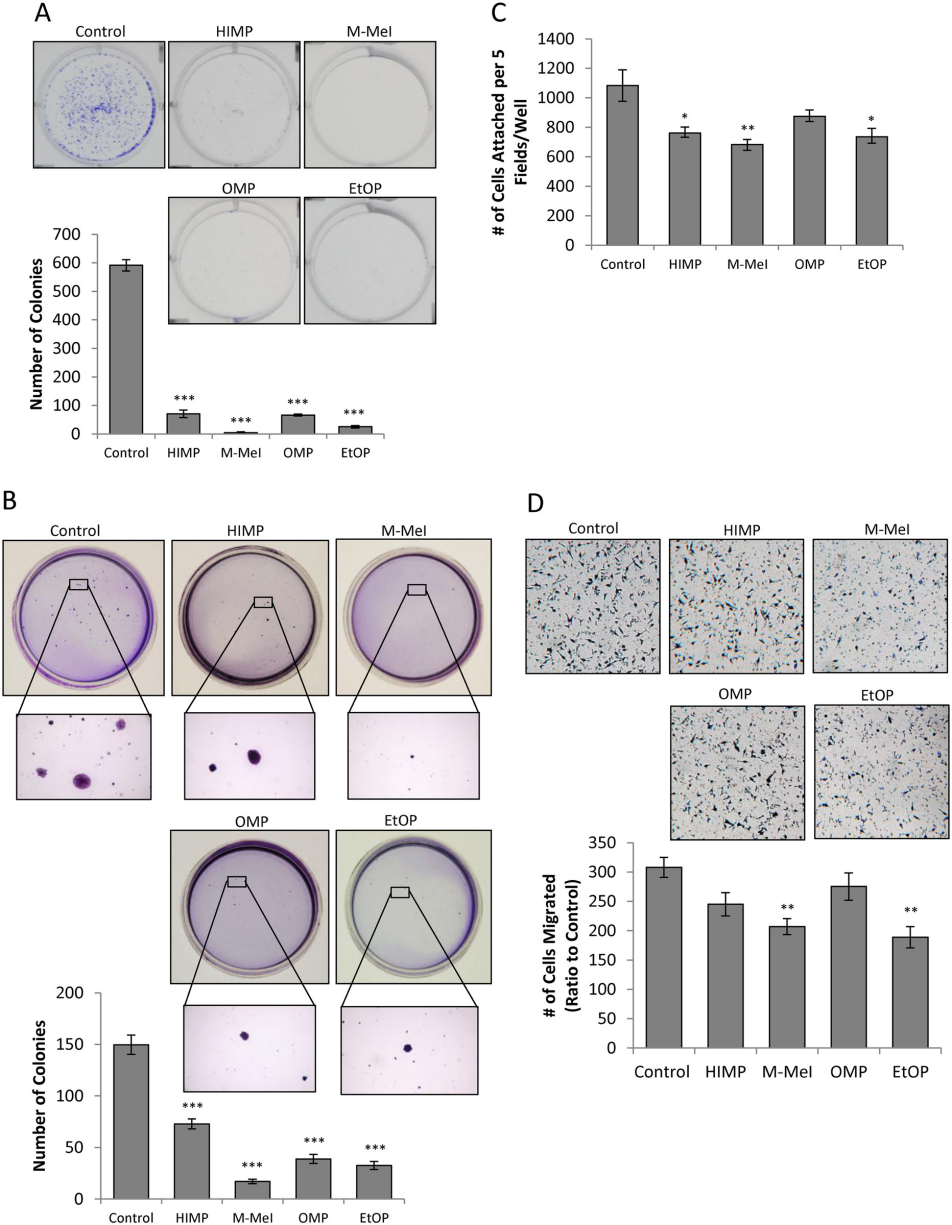
Effects of imidazopyridine derivatives on the tumorigenicity of LNCaP C-81 cells. **(A)** Clonogenic assay on plastic wares. LNCaP C-81 cells were plated in six-well plates at densities of 20, 200, and 2,000 cells/well. After 24 hours, attached cells were treated with respective compounds at 10 μM concentrations of imidazopyridine derivatives or solvent alone as control. Cells were fed on days 3, 6, and 9 with fresh culture media containing respective inhibitors. On day 10, cells were stained and the number of colonies counted. The photos of representative colony plates were taken from plates seeded with 2,000cells/well, and the number of colonies shown was counted also from plates seeded with 2,000cells/well. Minimal colony formation was observed at densities of 20 and 200 cells/well. Results presented are mean ± SE; n = 2x3. ***p<0.0001. **(B)** Anchorage-independent soft agar assay. LNCaP C-81 cells were plated at a density of 5 x 10^4^ cells/35mm dish in 0.25% soft agar plates. The following day, cells in doublets or greater were marked and excluded from the study. Media were added every three days, and at the end of 5 weeks, colonies formed were stained and counted. Representative images of colonies are shown (above) and the colony number was counted (below). The experiments were performed in duplicate with 3 sets of independent experiments. Results presented are mean ± SE; n = 2x3. *** p<0.0001. **(C)**. Cell adhesion assay on plastic wares. Cells were suspended in treatment media for 30 minutes before being plated in 6-well plates at 3 x10^3^ cells/cm^2^ using the same treatment media. Cells were allowed to adhere for one hour, fixed and stained by 0.2% crystal violet solution (50:50, water:MeOH). The total number of cells in five fields at 40x magnification for each well was counted. The experiments were performed in triplicate with 3 sets of independent experiments. Results presented are mean ± SE; n = 3x3. *p<0.05; **p<0.01. **(D)**. Cell migration transwell assay. Cell migration was assessed via Boyden chamber. An aliquot of 5 x 10^4^ C-81 cells was seeded in the insert of 24-well plates in media containing 10 μM respective compounds with solvent alone for control in both upper and lower chambers. After 24-hour incubation, the migrated cells were stained and those cells remaining in the upper chamber were removed via cotton swab. Cells which had migrated through to the lower chamber were counted. Representative images are shown at 40x magnification. The experiments were performed in triplicate with 3 sets of independent experiments, and the results presented are mean ± SE; n = 3x3, *p<0.05; **p<0.005.

The compounds’ ability to suppress colony formation in LNCaP C-81 cells was initially accessed by in vitro clonogenic assays for anchorage-dependent cell growth. LNCaP C-81 cells were seeded with 20, 200, and 2,000 cells per well in 6-well plates, and then treated with 10μM of each compound. Upon 10-days of treatment, all compounds significantly inhibited clonogenic growth at 2,000 cells per well as shown in Fig 3A for 2,000 cells per well. While M-MeI and EtOP strongly inhibited colony growth, HIMP and OMP were comparatively less potent. Minimal colony formation was observed at densities of 20 and 200 cells per well.

The soft agar colony formation assay was then performed to determine the compounds’ effect on anchorage-independent growth in a 3-dimentional environment. As shown in Fig 3B, cells cultured at a density of 5,000 cells per 35 mm dish for four weeks produced far fewer colonies with smaller colony size when treated with the imidazopyridine derivatives. Compared to control cells treated with solvent alone, M-MeI suppressed colony growth to the greatest extent, reducing the number of colonies by 90 percent with barely visible colony size (Fig 3B). In comparison, EtOP and OMP reduced colony growth with 80 and 70 percent inhibition, respectively, and HIMP had the least effect at about 50 percent inhibition.

To clarify whether these compounds’ effect on colony formation is in part due to the inhibition of cell adhesion, the capacity of HIMP, M-MeI, OMP, and EtOP to influence PCa cell adhesion onto the plastic surface of 6-well plates was then investigated. While these compounds had varying degrees of suppression on the ability of LNCaP C-81 cells to adhere at a density of 50,000 cells per well, a similar inhibitory trend was observed to that of clonogenic and soft agar assays (Fig 3C vs. 3A & 3B). M-MeI had the greatest effect and was able to reduce cell attachment by about 40 percent. While HIMP and EtOP were also able to significantly inhibit cell adherence, they did so to a lesser extent; OMP was found to have a minimal effect on the C-81 cells’ ability to attach to the plastic surface. Hence, though all compounds belong to the same class of molecules, they influence PCa cell colony formation and adhesion differently.

To investigate the inhibitory ability of these compounds on tumor metastasis, their activity on cell migration was analyzed by transwell migration assay. Interestingly, these compounds were found to have varying degrees of suppression on PCa cell migration. Fig 3D showed that both M-MeI and EtOP were able to significantly reduce LNCaP C-81 cell migration via Boyden Chamber assay over a period of 24 hours by about 30 percent. Comparatively, HIMP and OMP failed to significantly reduce cell migration. Overall, M-MeI was found to exhibit the most potent inhibitory activity on CR PCa cell tumorgenicity.

### Effect of Imidazopyridine Compounds on Proliferative and Apoptotic Signaling in CR PCa Cells

**Fig 4:**
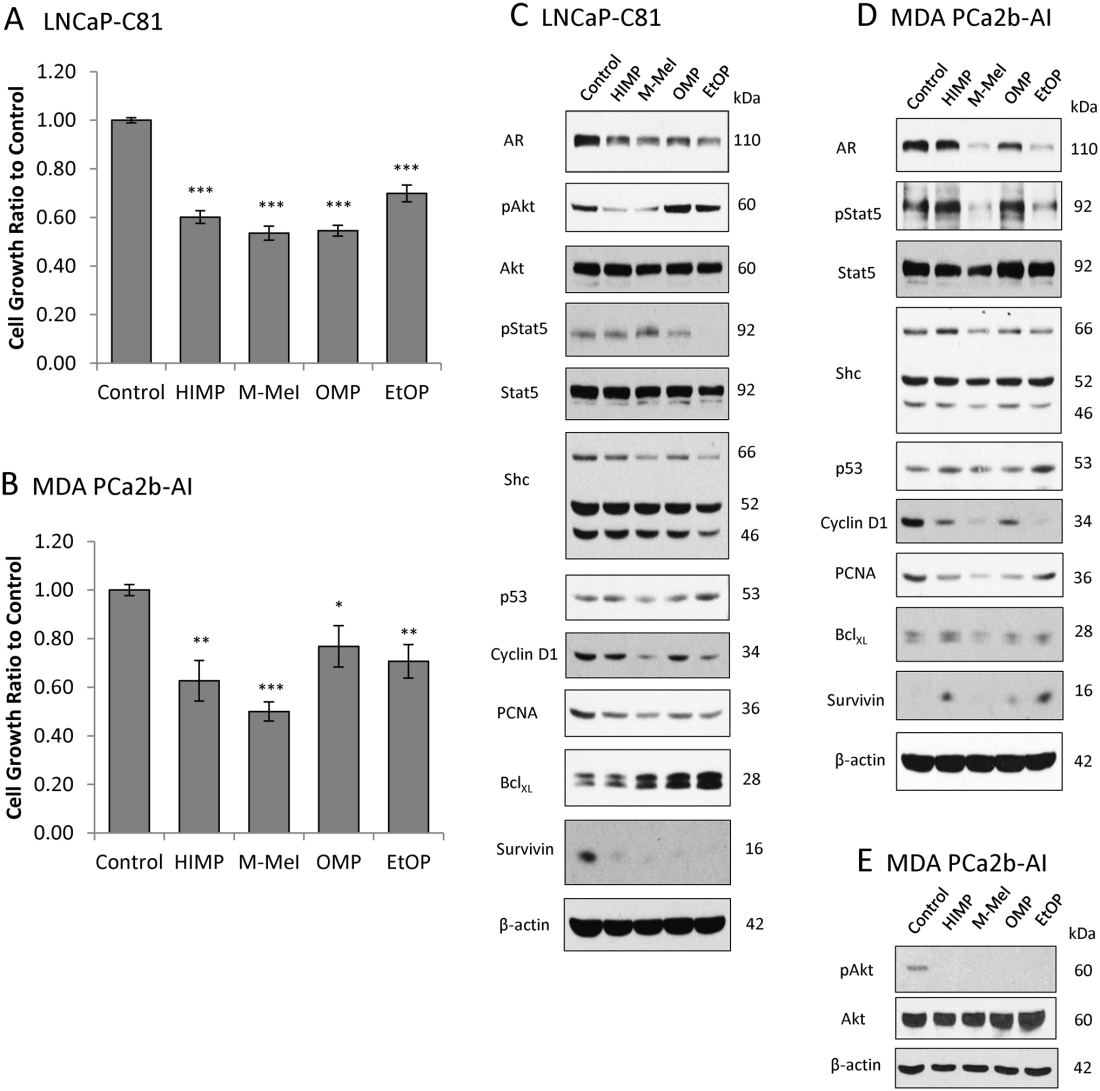
Effects of imidazopyridine derivatives on PCa proliferative and apoptotic signaling under SR conditions. **(A)** LNCaP C-81 cells were plated in triplicate in T25 flasks at 4 x 10^3^ cells/cm^2^ in regular medium, grown for 72 hours then steroid starved for 48 hours. Cells were then treated with 10 μM imidazopyridine derivatives or DMSO as control for an additional 72 hours under SR conditions. Cells were trypsinized and live cell numbers counted via Trypan Blue assay. The experiments were performed in triplicate with 3 sets of independent experiments. *** *p*<0.0005. **(B)** MDA PCa2b-AI cells were grown, treated, and counted under conditions as described above in (A) for LNCaP C-81 cells. Results presented are mean ± SE; n = 3x3. *p<0.05; **p<0.005; ***p<0.0005. **(C)** LNCaP C-81 total cell lysate proteins were collected from (A) after cell number counting. Those cells were grown in SR conditions and analyzed for phosphorylated Akt and STAT5, as well as total AR, Akt, Shc, p53, cyclin B_1_, cyclin D_1_, PCNA, Bcl_XL_, and Survivin protein levels. β-actin protein level was used as a loading control. Similar results were observed in two sets of independent experiments. **(D)** MDA PCa2b-AI total cell lysate proteins from (B) after cell number counting. Cells were grown in SR conditions and analyzed for phosphorylated STAT5, as well as total AR, Shc, p53, cyclin B_1_, cyclin D_1_, PCNA, Bcl_XL_, and Survivin protein levels. β-actin protein level was used as a loading control. Similar results were observed in three sets of independent experiments. **(E)** MDA PCa2b-AI total cell lysate proteins from cells grown in regular conditions were analyzed for phosphorylated Akt and total Akt. β-actin protein level was used as a loading control. Similar results were observed in three sets of independent experiments.

It is well established that the majority of CR PCa cells express functional AR which is still required for their growth and survival [22,31,32]. To determine how the compounds suppress PCa cell proliferation, we analyzed their effects on proliferative and apoptotic signaling in AR-positive LNCaP C-81 and MDA PCa2b-AI cells under SR conditions. Fig 4A and 4B showed that, upon 3-day treatments, 10 μM of each compound significantly suppressed LNCaP C-81 and MDA PCa2b-AI cell proliferation.

In LNCaP C-81 cells under SR conditions, though imidazopyridines are known for Akt inhibition, only HIMP and M-MeI inhibited Akt activation as shown by decreased Ser473 phosphorylation (Fig 4C) [15]. Additionally, while all compounds reduced AR protein levels in LNCaP C-81 cells; M-MeI and EtOP were more potent. Importantly, a similar trend was observed in AR-regulated pro-proliferative proteins. M-MeI and EtOP reduced levels of p66Shc, a 66kDa Src-homologous collagen homologue, cyclin D_1_, and PCNA, while HIMP and OMP had minimal effects [33–36]. We also analyzed Stat5 phosphorylation at Y694, which aids in the translocation of AR to the nucleus and is a regulator of cylcin D_1_ synthesis [37,38]. Unexpectedly, M-MeI slightly increased Stat5 activation in C-81 cells while EtOP suppressed activation; HIMP and OMP had no effect. Though all compounds diminished anti-apoptotic Survivin protein, their treatment elevated Bcl_XL_, another anti-apoptotic protein [39,40]. The compounds’ effect on p53, a regulator of cell survival and inducer of apoptosis, varied with M-MeI slightly lowering p53 levels, and EtOP slightly increasing them; HIMP and OMP had no significant effect [41].

As shown in Fig 4D, in MDA PCa2b-AI cells under SR conditions, AR inhibition was similar to that of LNCaP C-81 cells: M-MeI and EtOP greatly suppressed AR levels while HIMP and OMP had minimal effects. This also correlated with lower levels of AR-regulated p66Shc, cyclin D_1_, and PCNA in M-MeI-treated cells, all of which promote cell growth. As seen in C-81 cells, in MDA PCa2b-AI cells HIMP and OMP had no inhibitory effect on Stat5 activation, while EtOP suppressed Y695 phosphorylation. M-MeI, however, strongly reduced Stat5 phosphorylation in MDA PCa2b-AI cells where it had increased it in C-81 cells. Also, while the compounds’ effect on p53 remained similar in MDA-AI cells compared to C-81 cells, their effects on anti-apoptotic Survivin and Bcl_XL_ proteins were altered. In MDA-AI cells, HIMP, OMP and EtOP increased levels of Survivin while Bcl_XL_ remained unchanged relative to control cells suggesting these proteins are not essential to growth inhibition. Because Akt phosphorylation at Ser473 in MDA PCa2b-AI cells was undetectable under SR conditions, these cells were instead treated in regular culture medium for three days and all compounds were found to reduce Akt activation (Fig 4E).

In summary, of the four compounds tested, M-MeI was the most potent inhibitor of proliferation in both LNCaP C-81 and MDA PCa2b-AI cell lines under SR conditions. It also consistently reduced AR and AR-regulated proteins as well as Akt Ser473 phosphorylation in both cell lines, suggesting these pathways are involved in imidazopyridine inhibition of CR PCa cell growth.

### Kinetic Effect of HIMP and M-MeI on LNCaP C-81 Cells under SR Conditions

**Fig 5:**
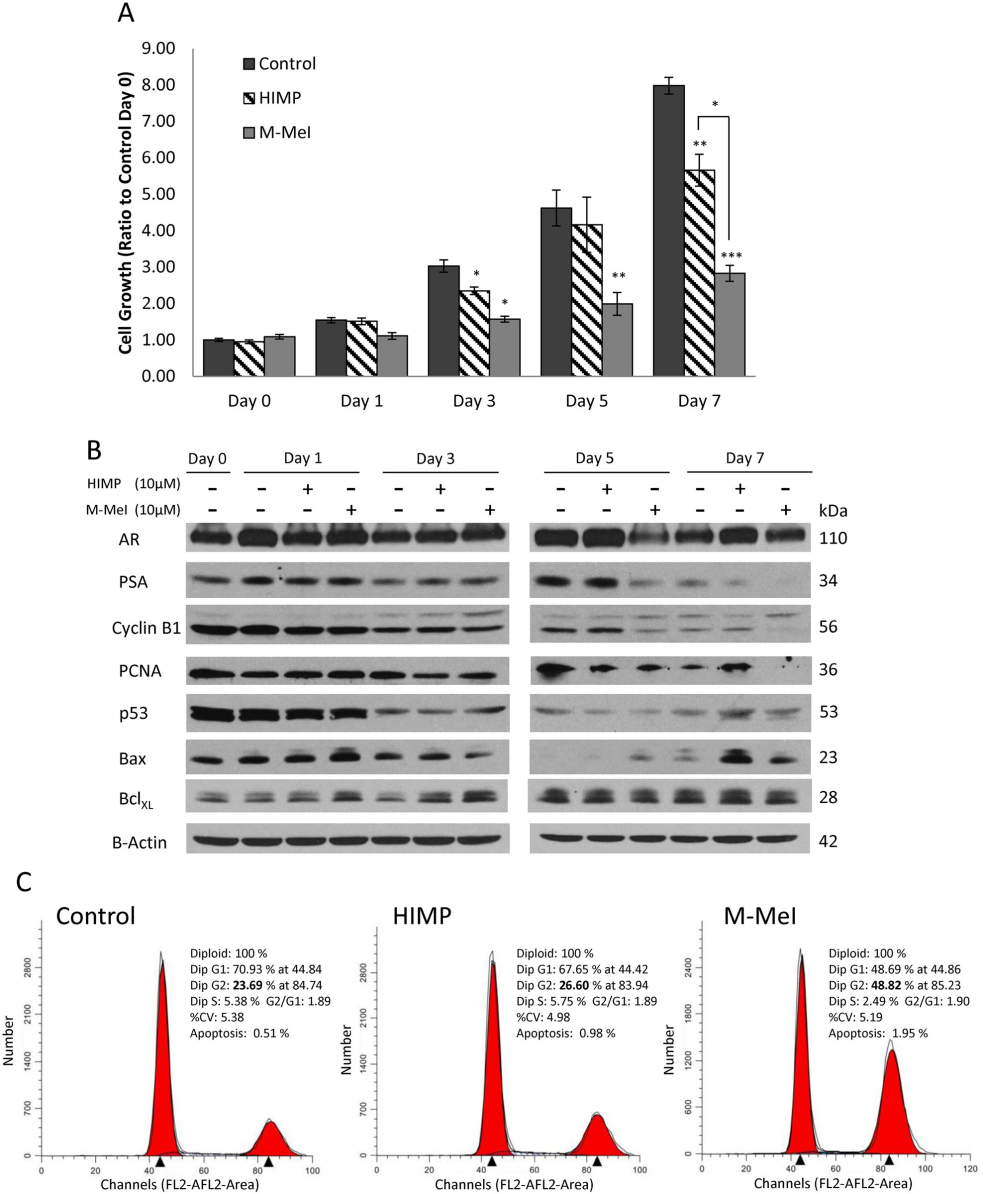
Kinetic analysis of HIMP and M-MeI’s effects on LNCaP C-81 cells under steroid-deprived conditions. **(A)** Cells were plated in six-well plates at 2 x 10^3^ cells/cm^2^ in regular medium for three days, then steroid-starved for 48 hours followed by treatment with 10 μM HIMP or M-MeI in SR medium containing 1 nM DHT. Solvent alone was used for controls. On day 0, 1, 3, 5 and 7, one set of cells in duplicates from each group was harvested for live cell counting. Remaining cells were replenished with fresh respective medium. The experiments were performed in duplicates with 3 sets of independent experiments. Results presented are mean ± SE; n = 2x3. *p<0.05; **p<0.005; ***p<0.0005. **(B)** Total cell lysate proteins from HIMP- and M-MeI-treated C-81 cells from (A) were collected and analyzed for AR, cPSA, cyclin B_1_, PCNA, Bax, p53, and Bcl-X_L_ proteins. β-actin protein level was used as a loading control. **(C)** Histograms of cell cycle distributions of LNCaP C-81 cells upon 7 days of HIMP and M-MeI treatments. Cells were plated in T25 flasks at 2 x 10^3^ cells/cm^2^ in regular medium for three days, then steroid-starved for 48 hours followed by treatment with 10 μM HIMP or M-MeI in SR medium with 1 nM DHT and solvent DMSO alone as control. One set of cells from each group was harvested after 3, 5, and 7 days treatment for flow cytometric analysis. Similar results were obtained from two sets of independent experiments. The data shown were representative results of 7-day treatments.

Since HIMP and M-MeI exhibited the most selectively potent activity, these two compounds were investigated further by kinetic analysis in LNCaP C-81 cells under SR conditions for clinical relevance. As shown in Fig 5A, both HIMP and M-MeI began to show significant inhibition of cell proliferation on day three of treatment, and this trend continued through day seven. It should also be noted M-MeI exhibited greater growth suppression than HIMP at every time point analyzed.

Cell lysates were collected from each time point and Western Blot analysis was performed (Fig 5B). Our results showed M-MeI-treated cells had decreased levels of AR and PSA, an androgen-regulated protein, as well as cell cycle proteins cyclin B_1_ and PCNA as seen at 5-day treatment. Meanwhile, HIMP treatment decreased PSA protein level upon 7-day treatment but not AR protein level. A similar trend was observed in pro-proliferation proteins as HIMP had no effect on cyclin B_1_ and marginal effect on PCNA. It should be noted, as the level of active unphosphorylated cyclin B_1_ decreased (lower band), the inactive phosphorylated cyclin B_1_ protein level increased (upper band) [42]. The data may indicate arrestment of the cell cycle.

We also investigated the effects of HIMP and M-MeI on pro-apoptotic proteins Bax as well as p53 vs. anti-apoptotic Bcl-X_L_ [40]. Treatment with both compounds dramatically increased Bax and p53 protein level by day seven and correlated with the observed decrease in cell proliferation. Interestingly, both compounds initially increased Bcl-X_L_ levels on days one and three, and had no effect on Bcl-X_L_ as treatment continued (Fig 4B). Thus both compounds seem to induce apoptosis by increasing levels of pro-apoptotic proteins instead of inhibiting anti-apoptotic proteins, while M-MeI also reduces pro-proliferative proteins as well as AR signaling.

To further investigate the kinetic effect of HIMP and M-MeI, cell cycle analysis was performed via flow cytometry. The cell cycle analyses (Fig 5C) revealed that upon 7-day treatment, M-MeI reduced LNCaP cell proliferation by 50% as indicated by the decreased percentage of cell population in S phase. M-MeI also increased the percentage of cells undergoing apoptosis nearly four-fold at day seven relative to control cells. At the same time, HIMP had no significant effect on percentage of cells in S phase and increased the percentage undergoing apoptosis two-fold by day seven. Interestingly, the population of cells treated with M-MeI, but not HIMP, accumulated in G2 phase following the time course of treatment, i.e., only a marginal increase on day 3 and about a 50% increase in day 5 (S1 Fig). On day-7, the percentage of cells in G2 phase doubled to about 49% in M-MeI treated cells compared to 24% in control cells (Fig 5C). In parallel, on day 7, the HIMP-treated cells were only slightly increased from about 24% to 27% in G2-phase. These results clearly indicate M-MeI blocks cell cycle at G2 phase.

## Discussion

Given the poor 5-year 29% survival rate of metastatic CR PCa patients, there is a clear need for advancement in treatment alternatives [14]. First line treatment of PCa usually involves ADT by means of surgical castration or chemical castration, such as luteinizing hormone-releasing hormone treatment, coupled with anti-androgens. Treatment with classic anti-androgens such as flutamide or bicalutamide (Casodex), which competitively inhibit androgen binding to AR, can be effective for 1–3 years before patients subsequently become unresponsive and eventually relapse. Relapse can occur for a number of reasons such as deregulation of AR cofactors, AR overexpression, splicing mutations resulting in constitutively active AR, or mutations allowing AR activation by competitive inhibitors [43]. In the case of the latter, upon discontinuation of flutamide or bicalutamide treatment, patients often experience a period of anti-androgen withdrawal syndrome (AAWS) characterized by a decline in serum PSA levels and tumor regression [43,44]. In some cases, these patients will respond to treatment with alternative anti-androgens, however, patients will again eventually become unresponsive to treatment and develop advanced PCa, commonly referred to as “castration-resistant” (CR) [45]. While more effective anti-androgens such as enzalutamide, which possesses a 5-fold higher binding affinity to AR compared to bicalutamide, are now available, mutations allowing AR activation by enzalutamide have been reported and tumors continue to become CR over time [46]. The therapeutic effect of second generation anti-androgens such as enzalutamide and abiraterone acetate implies most cancer cells, which still express functional AR, require AR signaling to evade traditional regulatory mechanisms to survive androgen deprivation strategies [7,8,47]. Despite the effectiveness of ADT and anti-androgen treatment strategies to delay the progression of PCa, many patients still develop the CR phenotype and thus there is an urgent need for alternative therapeutic targets to AR. Other signaling pathways, such as the PI3K/Akt-mediated cell survival pathway, may act to supplement the lack of AR stimulation under androgen-deprived conditions, bypassing the effect of ADT. Therefore, in this study we investigate novel therapeutic agents which are capable of targeting multiple biochemical pathways critical to CR PCa cell growth and progression.

In this study, four novel imidazopyridine derivatives were investigated to determine their viability as therapeutic agents for CR PCa. LNCaP C-81 cells were chosen as our primary experimental cell model because they possess many biochemical properties common to CR PCa, including expression of functional AR, AI PSA secretion and proliferation, and expression of enzymes required for the synthesis of androgens from cholesterol [19,21,22,31,47]. We initially showed that all four compounds are effective inhibitors of CR PCa cell growth in the LNCaP C-81 cell line model, though to varying degrees with M-MeI as the most potent under both regular and SR conditions (Fig 2A). Additionally, the effects of the imidazopyridine derivatives on cell proliferation differed between compounds as well as cell lines. Of those compounds investigated, HIMP and M-MeI displayed broad-spectrum growth inhibition of multiple CR PCa cell lines, including both AR-positive and AR-negative cells. Most importantly, these compounds exhibited more potent inhibition of proliferation in PCa cells than benign RWPE1 cells (Fig 2B).

We further investigated the effects of each compound on various biological activities critical to malignant tumor progression. Colony formation, cell adhesion, and migration are vital malignant processes exhibited by many cancer cells. In both anchorage-dependent and anchorage-independent growth assays, M-MeI and EtOP dramatically reduced the number of colonies formed as well as colony size. This may partially be due to M-MeI and EtOP’s ability to inhibit cell adhesion. To investigate the effect of these compounds on cell migration, the transwell migration assay was used to analyze LNCaP C-81 cell motility. Again, M-MeI and EtOP were found to be potent inhibitors of C-81 cell migration while HIMP and OMP had no significant effect. Interestingly, this correlates with M-MeI and EtOP’s ability to reduce AR protein level as shown in Fig 4C where HIMP and OMP fail to influence AR. This suggests AR inhibition may be crucial for the inhibition of these malignant processes. However, while EtOP was an effective inhibitor of PCa tumorgenicity, it was not as effective at growth suppression as M-MeI. This may be due to EtOP’s failure to prevent Akt activation whereas M-MeI successfully inhibits Akt phosphorylation at S473 (Fig 4C).

The PI3K/Akt signaling pathway is proposed to be pivotal to the growth and survival of CR PCa cells; dysregulation of this pathway is shown to contribute to resistance to treatment [31,48–54]. In clinical studies, for example, treatment with AR inhibitor bicalutamide is shown to progressively increase Akt signaling in patients in correlation with their Gleason grades [51]. In parallel, PCa treated with chemotherapeutic agent docetaxel possess increased Akt activation in patient tumors [52]. Other *in vitro* studies similarly demonstrated LNCaP cells grown in SR medium for extended periods of time have increased Akt activation which may compensate for a lack of androgen signaling [12]. Indeed, there is high prevalence of PI3K/Akt/mTOR pathway activation in CR PCa and emerging studies show inhibitors targeting the PI3K/Akt pathway are rapidly entering into clinical trials [14,53–55]. Therefore, the PI3K/Akt signaling axis is a promising next-in-line therapeutic target and its inhibition in conjunction with ADT and anti-androgens may improve patient survival.

Initially, imidazopyridines have been shown to possess Akt kinase inhibitory activity and are effective suppressors of tumor growth and advancement in a number of carcinomas, including PCa [15,16,18]. Interestingly, while all four derivative compounds inhibited Akt phosphorylation at Ser473 in MDA PCa2b cells; only HIMP and M-MeI inhibited its phosphorylation in LNCaP C-81 cells. It should be noted because Akt signaling in MDA PCa2b-AI cells was too weak to detect under SR conditions, it was observed under regular conditions. It is thus possible the differential inhibition of Akt activation in MDA-AI vs. LNCaP C-81 cells was in part due to the difference between regular and SR growth conditions (Fig 4C–4E).

While HIMP is a strong inhibitor of Akt activation in both cells, it fails to consistently down-regulate AR, a long established target of PCa therapy. Supportively, western blot analysis showed HIMP inconsistently affects AR protein levels (Figs 4D and 5B), a phenomena which has previously been observed when CR PCa cells are treated with PI3K/Akt inhibitors [12,18]. Furthermore, HIMP had little shift in potency between regular conditions and SR conditions (Fig 2A), suggesting HIMP’s mechanism of inhibition is relatively androgen-independent. In addition, HIMP is a potent inhibitor of Akt phosphorylation at Ser473 and also acts to induce pro-apoptotic p53 and Bax proteins (Figs 4C, 4D and 5B) [27]. Together, these results suggest HIMP inhibits CR PCa growth by suppressing cell survivability but not androgen signaling.

While both HIMP and M-MeI were found to have promising selective growth suppression, M-MeI was shown to have greater efficacy (Figs 2 and 4). M-MeI is a derivative of HIMP, possessing a methyl group on the para position of the benzene ring (Fig 1). This modification apparently allows M-MeI to suppress AR protein level as well as Akt activation; there is also a noticeable change in M-MeI’s IC_50_ value between regular and SR conditions, indicating its mechanism of growth inhibition includes the suppression of AR signaling (Fig 4C–4E). Supportively, as shown in Fig 5B, upon M-MeI treatment, there is a consistent, progressive decrease in AR protein. This coincides with a decrease in PSA, a target of AR, as well as p66Shc protein level, a protein regulated by androgens which is involved in cell proliferation and apoptosis (Fig 4C and 4D). Downstream pro-proliferative markers, cyclin D_1_ and PCNA were also down-regulated over-time to a greater extent by M-MeI compared to HIMP. In addition, STAT5 signaling is proposed to be involved in the transition from androgen-sensitive to CR PCa and activated STAT5 can enhance nuclear translocation of AR [40]. Interestingly, as seen in Fig 4C and 4D, while M-MeI inhibited Stat5 phosphorylation at Y694 in MDA-AI cells, it increased Y694 phosphorylation in LNCaP cells. This inconsistent effect suggests inhibition of STAT5 activation is not vital to M-MeI’s suppression of PCa. Furthermore, M-MeI strongly inhibited the phosphorylation of pro-survival protein Akt (Ser473) (Fig 4C–4E) as well as induced pro-apoptotic proteins p53 and Bax (Fig 5B) [27]. Thus, M-MeI’s anti-tumorigenic effect on PCa is due to its strong inhibition of both Akt and AR signaling pathways.

Unexpectedly, the kinetic analysis revealed an interesting phenomenon: upon HIMP and M-MeI treatments over time, both compounds, though to a greater extent by M-MeI, decreased the protein level of active unphosphorylated cyclin B_1_ (Fig 5B, lower band) and increased the inactive phosphorylated band (upper band) (Fig 5B) [41]. Cyclin B_1_, a key regulator of mitosis, is phosphorylated during the G2 phase and becomes unphosphorylated upon the cell reaching M phase [41]. To validate further, we performed cell cycle analysis on HIMP- and M-MeI-treated cells by flow cytometry. Compared to control cells, M-MeI-treated cells had lower percentages of cells in S phase, corresponding with a decrease in cell proliferation, and twice the percentage of cells in the G2 phase. Minimal changes were observed in HIMP-treated cells, although both treatments increased the percentage of cells undergoing apoptosis (Fig 5C). Together, this data indicates these imidazopyridine derivatives function to inhibit the transition of PCa cells between G2 and M phases, though the exact mechanism remains to be elucidated.

In summary, our data shows imidazopyridine derivatives exhibit inhibitory activity of tumorgenicity in CR PCa cells. Each compound displayed differential effects on Akt and AR signaling pathways, and further studies are needed to determine the mechanism by which these molecules suppress the growth of CR PCa. Among the four compounds investigated, M-MeI was found to suppress multiple signaling pathways related to PCa progression, including classical target AR as well as the Akt survival pathway, making it a promising candidate for future therapeutic studies. Importantly, this compound displays selective growth inhibition, having a significantly greater suppressive effect on PCa compared to benign prostate epithelial cells. Thus, M-MeI can serve as a lead compound for imidazopyridine side-chain modifications, which could yield more potent, selective agents to improve the treatment of CR PCa patients. Future investigation to elucidate M-MeI’s specific mechanism of inhibition and *in vivo* studies will help better determine its potential as a therapeutic agent.

## Supporting Information

S1 Fig Histograms of cell cycle distributions of LNCaP C-81 cells upon 3 and 5 days of HIMP and M-MeI treatments. Cells were plated in T25 flasks at 2 x 10^3^ cells/cm^2^ in regular medium for three days, then steroid-starved for 48 hours followed by treatment with 10 μM HIMP or M-MeI in SR medium with 1 nM DHT and solvent DMSO alone as control. One set of cells from each group was harvested after 3, 5, and 7 days treatment for flow cytometric analysis. Similar results were obtained from two sets of independent experiments. The data shown were representative results of 3- and 5-day treatments.
(TIF)
