# Supplementary figures and images for: Novel Imidazopyridine Derivatives Possess Anti-Tumor Effect on Human Castration-Resistant Prostate Cancer Cells
Source: PLoS One. 2015 Jun 29;10(6):e0131811. doi: 10.1371/journal.pone.0131811 (PMC4487901; doi:10.1371/journal.pone.0131811)

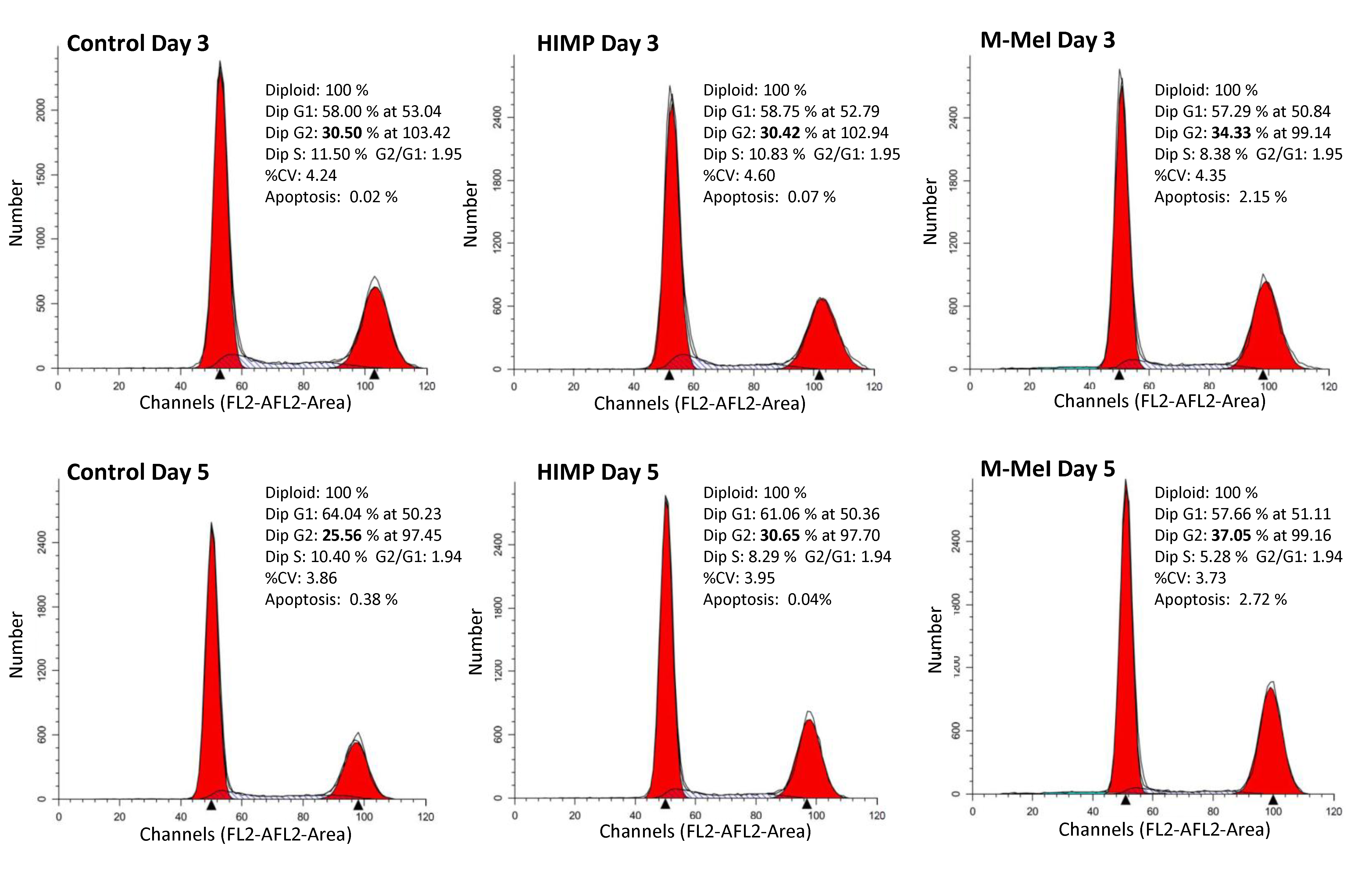

Supplement: S1 Fig — Cells were plated in T25 flasks at 2 x 10^3^ cells/cm2 in regular medium for three days, then steroid-starved for 48 hours followed by treatment with 10 μM HIMP or M-MeI in SR medium with 1 nM DHT and solvent DMSO alone as control. One set of cells from each group was harvested after 3, 5, and 7 days treatment for flow cytometric analysis. Similar results were obtained from two sets of independent experiments. The data shown were representative results of 3- and 5-day treatments. (TIF) [file pone.0131811.s001.tif]
